# Computational kinematics of dance: distinguishing hip hop genres

**DOI:** 10.3389/frobt.2024.1295308

**Published:** 2024-05-02

**Authors:** Ben Baker, Tony Liu, Jordan Matelsky, Felipe Parodi, Brett Mensh, John W. Krakauer, Konrad Kording

**Affiliations:** ^1^ Davis AI Institute, Department of Philosophy, Colby College, Waterville, ME, United States; ^2^ Department of Computer and Information Sciences, University of Pennsylvania, Philadelphia, PA, United States; ^3^ Applied Physics Laboratory, Johns Hopkins University, Laurel, MD, United States; ^4^ Department of Bioengineering, University of Pennsylvania, Philadelphia, PA, United States; ^5^ Department of Neuroscience, University of Pennsylvania, Philadelphia, PA, United States; ^6^ Janelia Research Campus, Howard Hughes Medical Institute, Ashburn, VA, United States; ^7^ Departments of Neurology, Neuroscience, Physical Medicine and Rehabilitation, Johns Hopkins University, Laurel, MD, United States; ^8^ Santa Fe Institute, Santa Fe, NM, United States

**Keywords:** dance, movement analysis, machine learning, genre classification, hip hop dance, dance perception, movement representation

## Abstract

Dance plays a vital role in human societies across time and culture, with different communities having invented different systems for artistic expression through movement (genres). Differences between genres can be described by experts in words and movements, but these descriptions can only be appreciated by people with certain background abilities. Existing dance notation schemes could be applied to describe genre-differences, however they fall substantially short of being able to capture the important details of movement across a wide spectrum of genres. Our knowledge and practice around dance would benefit from a general, quantitative and human-understandable method of characterizing meaningful differences between aspects of any dance style; a computational kinematics of dance. Here we introduce and apply a novel system for encoding bodily movement as 17 macroscopic, interpretable features, such as expandedness of the body or the frequency of sharp movements. We use this encoding to analyze Hip Hop Dance genres, in part by building a low-cost machine-learning classifier that distinguishes genre with high accuracy. Our study relies on an open dataset (AIST++) of pose-sequences from dancers instructed to perform one of ten Hip Hop genres, such as Breakdance, Popping, or Krump. For comparison we evaluate moderately experienced human observers at discerning these sequence’s genres from movements alone (38% where chance = 10%). The performance of a baseline, Ridge classifier model was fair (48%) and that of the model resulting from our automated machine learning pipeline was strong (76%). This indicates that the selected features represent important dimensions of movement for the expression of the attitudes, stories, and aesthetic values manifested in these dance forms. Our study offers a new window into significant relations of similarity and difference between the genres studied. Given the rich, complex, and culturally shaped nature of these genres, the interpretability of our features, and the lightweight techniques used, our approach has significant potential for generalization to other movement domains and movement-related applications.

## 1 Introduction

The way we move our bodies is abundant with meaning. Slightly tilting one’s head or shifting one’s feet can speak volumes. Some of these connotations are intentional, some are specific to culture or locality, and some arise from prehistoric aspects of our embodied activity in the world. Expressive movement is perhaps most vivid in dance, where it is sculpted, explored, and composed to artistic ends. The complex and culturally shaped nature of dance presents a challenge for formal analysis. Although experts develop an implicit understanding of dance styles, and some can verbalize components of style, the work developing effective quantitative approaches has been highly limited. There are notation schemes to record dance movement, such as Labanotation and Benesh notation, which have served as important tools, especially for documentation of classical western styles (Guest and Anderson, 1970). Some researchers in robotics have developed quantitative means to represent abstract elements of dance, drawing on labanotation or focusing on a specific form of dance, like Ballet (LaViers et al., 2011; El Raheb and Ioannidis, 2013; Pakrasi et al., 2018). However, these approaches are substantially restricted in their precision and generalizability. To our knowledge there has been no algorithmically implementable description of full-bodied movement that captures significant aspects of movement across diverse dance genres. Here we introduce and examine a system designed to fill this gap–a computational kinematics. By applying our system to the richly textured realm of Hip Hop Dance, we uncover readily interpretable aspects of movement that make a difference to the attitude or aesthetic of a Hip Hop dancer. We also shed new light on the characteristics of these genres and the relations between them.

Dance genres emerge as unique expressions of different communities, and the socio-historical context surrounding modern Hip Hop genres make them a crucial subject of study (New Cultural Studies of Dance, 1997; Thomas, 2003; Bennett, 2022). Hip Hop originates in Black and LatinX communities in New York City in the 70s, and is rooted in afro-diasporic forms of music and movement (Rivera, 2003; Chang, 2005; Schloss, 2009; Charnas, 2010; Morgan and Bennett, 2011; Durden, 2022). Hip Hop quickly transcended its initial birthplace, proliferating globally through media technologies by the 80s. Television shows such as “Soul Train” and films like “Breakin” brought Hip Hop to international audiences. Elite dancers and groups like Rock Steady Crew and the Electric Boogaloos started traveling to share their craft and compete in international competitions, and later the internet greatly amplified Hip Hop’s renown. This globalization has been supported by Hip Hop’s entwinement with commercial sectors, notably entertainment and fashion, which have frequently adopted and contributed to a Hip Hop aesthetic (Osumare, 2007; Bennett, 2022). Hip Hop Dance culture emphasizes inclusivity and community, steering away from a need for formal training and prestigious venues, and instead invites dancers to converse with their neighbors and family and distinguish themselves through unique characters of movement. The dance form serves as a vehicle for these communities to express solidarity and resistance in the face of institutional marginalization and stigmatization, and the perspectives embodied in Hip Hop movement are often sidelined in scientific and technological investigations. Given its participatory ethos, its emphasis on individual style, and the way it is simultaneously tied to a local setting and to commerce, Hip Hop Dance has undergone rapid and extensive “genrefication” on street corners and stages almost everywhere in the world. This makes Hip Hop Dance an ideal subject of study in which to explore a new, computational approach to developing knowledge around expressive movement.

The present moment offers great potential to leverage advances in computational tools to further our understanding of dance and movement. New opportunities have arisen due to significant advances in pose estimation and machine learning techniques (Wang et al., 2021; Wang and Yan, 2023), as well as the compilation of large and freely available movement datasets, including datasets of dance sequences labeled according to genre (Castro et al., 2018; Li et al., 2021). There are, however, substantial challenges to extracting meaningful qualities of dance using these tools. Consider that a genre might stand out as being relatively communal as opposed to confrontational, as House compares to Breakdancing, or as accessible as opposed to uncanny, as LA Hip Hop compares to Krump (DeFrantz, 2022). These are qualities of dance forms that can be untangled from contextual elements like the music or setting, so they should have reliable forms of presentation in movement data. Yet they unfold through changing dynamics across many parts of the body and do not relate straightforwardly to the change of pose between two frames of a video. While audiences can often sense abstract features of a movement, substantial expertise is required to describe them in any detail. To formalize these differences in mathematical functions of frame-to-frame changes in joint-position calls for a fusion of knowledge in computational techniques and in dance.

**FIGURE 1:**
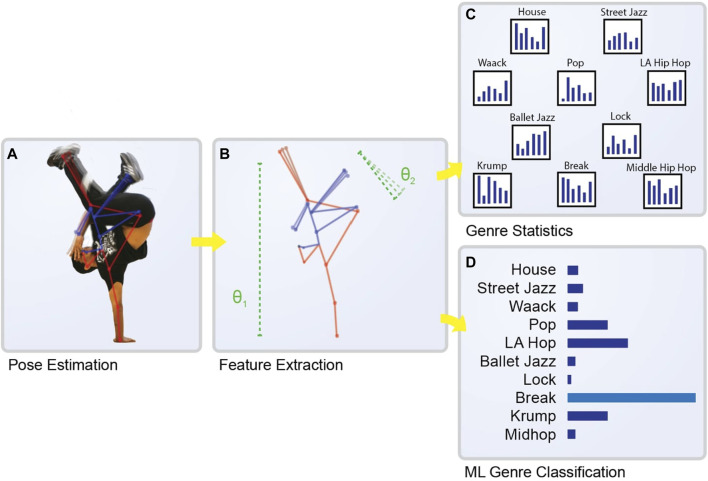
Overview of our machine learning pipeline for classifying Hip Hop dance genre. **(A)** The first stage involves the extraction of pose data from the dancer’s performance, visualized here with a skeletal pose overlaid on a dancer mid-routine **(B)** This raw pose data is then transformed into a set of high-level features, represented here by the dotted lines and symbols **(C)** The genres in the dataset are compared in terms of the features **(D)** These features are used by the machine learning classifier to make a prediction about the genre of the dance, here visualized by a bar plot representing the model’s probability assessments and a prediction for the genre. Images of dancers provided by TheFlavorContinues.org.

We introduce a novel system of encoding and analyzing human movement, apply our method in an analysis of ten Hip Hop Dance genres, and use it to equip a machine learning system to distinguish among these genres to a high degree of accuracy (Figure 1). Drawing on our personal experience with dance and human movement, we develop a set of 17 human-understandable movement features, such as bodily expansion, the lifting of the ankles, or how often there are sharp movements. We extract these features from sequences in the AIST++ dataset (Li et al., 2021), and train a machine learning classifier to recognize genre from this movement data (using no auditory information). We provide statistical analyses of the features in this data, evaluate the performance of our classifier along several metrics, and examine the influence of various features on the model’s classifications.

In order to offer a comparison to our automated system, we also investigate human perception of these genres of movement. There have been studies of the human capacity to distinguish various abstract categories from minimal displays of movement, including emotion, gender, and specific individuals (Johansson, 1973; Dittrich et al., 1996; Brownlow et al., 1997; Sevdalis and Keller, 2012). In some cases humans can very quickly identify the genre of a dance, but this ability varies considerably with experience and genre (Calvo-Merino et al., 2005). We conducted an online experiment where subjects were presented with clips of simplified, stick-figure renditions of dances from the dataset, and were asked to identify the dance genre. We have left the experiment online at https://genrejudge.experiments.kordinglab.com/, where the reader can experience the task firsthand.

## 2 Related work

Existing approaches to movement classification fall into two categories; those that rely principally on deep learning systems to find movement features that serve the classification task, and those that rely on handcrafted movement features (Wang and Yan, 2023), (Pareek and Thakkar, 2021). Handcrafted approaches have an advantage in computational costs and in the interpretability of models, but deep learning approaches have tended to outperform them in recent years and have become standard.

Handcrafted features used in prior research have captured low-level aspects of movement, focusing in on local motion at the scale of individual pixels or specific keypoints. For example, one prevalent method is optical flow, which represents the pattern of motion between two consecutive frames in terms of the direction and speed of apparent motion at each pixel. Other techniques include relying on a histogram of motion gradients (HOG), using the change in intensity or color between adjacent pixels to capture the orientation and magnitude of local movements across the body, or relying on the covariance of motion between every pair of joints on the body. Each of these techniques essentially dissect movement into many local measurements, resulting in long feature vectors (typically hundreds of values per frame) that can be used in a variety of computational classification methods.

Learned features are extracted through deep learning algorithms, and have become increasingly popular in Human Action Recognition (HAR). The input to these networks is a per-frame, detailed body representation, obtained from the pose-estimation method of choice, or sometimes from the kind of handcrafted measures described above. A commonly employed model is the Skinned Multi-Person Linear (SMPL) model, which creates a detailed mesh covering the depicted human body’s shape using millions of vertices (Loper et al., 2015). Recent approaches have gone beyond looking within each frame, using relationships across frames to derive temporal features. For instance, Spatial Temporal Graph Convolutional Networks (ST-GCN) can capture dynamic patterns of skeletal movement, providing a helpful framework for recognizing human actions (Yan et al., 2018). Another recent technique is PoseConv3D, which builds on this approach by representing human skeletons as a 3D heatmap volume instead of a graph sequence, enhancing the robustness against pose estimation noise (Duan et al., 2022). These approaches incorporate temporal information and leverage deep learning, providing powerful means for classifying complex movements, although they often come with high computational demands and challenges to interpretability.

The computational genre-classification of dance is a relatively new Frontier in the field of human action classification, where only a few have offered ways of modeling differences among dance styles. One such effort is a study exploring the influence of musical genre on improvised movement (Carlson et al., 2020). These researchers used music labeled according to genre as stimuli, prompted participants to dance freely, and used machine learning to predict musical genre. Their model performed above chance at 23.5% for an 8-class problem (chance equals 12.5%). Notably, the model was substantially better at identifying individual dancers than it was at discerning musical genres, suggesting idiosyncrasies in a specific person’s movement style are easier to pick out than the marks of a genre, at least for prevailing techniques. Another study, which is closest to our own in that it explicitly looks at genres of dance movement, utilized the “Let’s Dance” dataset, consisting of 1000 videos of dances from 10 genres from a broad range of historical and cultural settings (Castro et al., 2018). The best performing approach in this study uses RGB image data (for each frame) and optical flow data (between frames) as inputs into a convolutional neural network (CNN). This method demonstrated a notable level of success, with genre classification accuracy reaching around 70% in the best cases. As compared to the AIST++ dataset we rely on, the “Let’s Dance” dataset is made up of shorter (10 s) sequences, contains only 2D data, and includes genres that are more socio-historically distinct and adhere to stricter movement conventions, making them easier to distinguish by a naive audience. These studies highlight the ongoing challenges and progress toward computational modeling of the aspects of human movement that characterize dance genres.

The AIST++ dataset is one of the largest and richest public datasets of 3D joint positions from complex human movement, and the only such dataset comprising labeled Hip Hop Dance genres. This dataset has spurred exciting developments in the computational generation of dance sequences conditioned on the genres of AIST++ (Li et al., 2021), (Siyao et al., 2022; Tseng et al., 2022). The approach from Li et al.‘s “AI Choreographer” relies on a transformer-based model, which is trained to predict future frames of motion based on genre. This model utilizes separate transformers for both motion and music information, each labeled according to genre. A cross-modal transformer is employed to learn the correspondences between the embeddings of motion and music. Their system generates artificial motions that were judged more musically appropriate than three other approaches used as baselines. Siyao et al. and Tseng et al. further improve upon the motion-and-music processing models, outperforming Li et al. in user studies. They use a transformer-based diffusion model for motion, using auxiliary losses to enhance kinematic realism, and supplementing it with a devoted tool for extracting musical features. Siyao et al. introduce a new component called a ‘choreographic memory’, implemented by a Vector Quantized-Variational AutoEncoder (VQ-VAE). This enables their system to learn and recall common dance positions, contributing to the generation of dance movements that more closely match human styles. Collectively these studies have taken innovative strides in generating novel, musically apt, human-like dance movements. However they require sophisticated, high-computational-cost techniques involving deep neural networks and large feature vectors. In contrast, our work presents a more sparse yet potent method, leveraging a curated set of 17 human-understandable features to classify and analyze dance genres. Our methods are low-cost and provide readily interpretable results, allowing for greater understanding of aspects of expressive movements in Hip Hop Dance. Given the rich, complex, and culturally shaped nature of the differentiation of Hip Hop Dance genres, our approach has great potential for generalization to other movement domains and movement-related applications.

## 3 Methods

### 3.1 Data

We use joint-position sequences from different dance genres collected in the AIST++ Dance Motion Dataset. This dataset was constructed from the AIST Dance Video Database, and contains 1,408 sequences of 3D human dance motion captured at 60 frames-per-second (Tsuchida, 2023), (Li et al., 2021). These dance sequences span a duration range from 7.4 s to 48.0 s, and are equally distributed across ten dance genres: Ballet Jazz, Breakdance, House, Krump, LA Hip Hop, Lock, Middle Hip Hop, Pop, Street Jazz, and Waack. The sequences represent a variety of movements within each genre and total over 5 hours of dance footage. This dataset constitutes one of the largest and richest publicly available collections of 3D keypoints from complex human movement, and the only such collection of Hip Hop movement labeled by genre.

Importantly, the keypoint data is divided into “Basic” and “Advanced” sequences. Basic sequences average 9.25 s in length (SD 1.6), and tend to depict standard dance movements that repeat several times. Advanced sequences average around 36.5 s (SD 6.1) and are more dynamic and individualized. There are many more Basic than Advanced sequences; approximately 1200 and 200, respectively. We felt that it would be most interesting to test genre classification on the Advanced pieces, because a more general representation of genre is required to recognize the longer and more variable sequences. Further, given a training set composed almost entirely of Basic sequences (>90%), our model must be capable of some generalization in order to succeed on a test set composed entirely of Advanced pieces. Therefore a solution to the genre-classification task, posed on this split of this dataset, demands a versatile method of processing complex movement data.

### 3.2 Features

Having extensively participated in, observed, and discussed various dance genres, we sought to encode high-level aspects of human motion that make movements visually and kinesthetically distinct from one another. To extract the features we developed a pipeline that processes 3D joint position data and outputs a vectorized dance sequence. The pipeline first derives some essential measures from the raw data: it approximates the sacrum’s position and calculates the first three derivatives of position—velocity, acceleration, and jerk—for each joint. A Savitzky-Golay filter (Savitzky and Golay, 1964) was employed to smooth these derived measures, reducing noise while preserving the overall trends. These computations laid the foundation for the extraction of our 17 features into a vector representation of the dance sequence. Our method is flexible enough to accommodate a variety of joint data arrays and the features can be straightforwardly refined or augmented, making it adaptable for various investigations of dance and movement.

The feature we used can be divided into four broad categories: (1) movement of the sacrum, (2) movement of the extremities, (3) global angular momentum centered at the sacrum, (4) how expanded the body is from the sacrum.

The sacrum is situated at the base of the spine, and plays a pivotal role in the mechanics of all full-body movements. Forces must traverse the spine in order to direct the motion of the rest of the body, meaning large differences in degree or type of overall movement are reflected in sacral movement. The sacrum also provides for a simple approximation of the degree of translational motion in the XZ plane and the rising and falling of a dance in the Y dimension. The AIST++ dataset uses the common COCO human pose format, which omits the sacrum (and the rest of the spine). To overcome this, we approximate the sacrum’s position by averaging the coordinates of the hip joints. From this, we extract several measures of sacral movement, enabling us to capture essential dynamics of the dance.

The wrists and ankles play a crucial role in the overall impression and execution of a dance. Patterns of movement of the wrists contribute substantially to the feeling of the dance, as in when they flare out energetically, or lazily trail through space. The ankles serve as the point of contact with the ground, and therefore significantly reflect how a dancer’s weight is distributed. The extent and speed with which ankles lift away from the ground can distinguish between styles that emphasize light, leaping movements, or staccato footwork, or stability and groundedness. By tracking these extremities, we capture vital components of the dancer’s relationship with space and rhythm.

Angular momentum around the sacrum reflects the degree to which a dancer engages in turning or twisting motion. Dance is replete with rotation, and this comes in many shades—fast, slow, rising, falling, drawing inward (centripetal), or spiraling outward (centrifugal). Moreover, sharp dance movements translate into peaks in the trace of angular momentum over time, and these peaks can be counted to provide a measure of how much sharp movement a dance contains and in which directions.

Finally, an important aspect of the way a dancer uses space is a matter of how expanded or contracted their body is, and how this changes throughout the piece. For instance, wide, open movements can connote joy, strength, or freedom, while tightly contained movements can evoke introspection, restraint, or sorrow. Of course these kinds of expressions vary across context, but nonetheless a dancer’s expandedness corresponds significantly to the overall style of their dance.

Within each of the above categories of movement features we derived between two and six related measures, resulting in a total feature vector length of 17. Thus we arrive at a low-dimensional representation of a pose-sequence, where each feature speaks directly to an abstract but intuitive aspect of bodily movement.

Here we present mathematical expressions for a representative subset of the features. The remaining features can be straightforwardly derived from these expressions.

Letting *SP*_*i*_ represent the 3D sacrum position at the *i*th frame of a sequence (and given a constant interval between frames) finite differences can be used to estimate the first three derivatives of motion S*V*_*i*_ (velocity), S*A*_*i*_ (acceleration) and S*J*_*i*_ (jerk) as follows:

$${SV}_{i}=\frac{{SP}_{i+1}-{SP}_{i}}{\Delta T}$$

$${SA}_{i}=\frac{{SV}_{i+1}-{SV}_{i}}{\Delta T}$$

$${SJ}_{i}=\frac{{SA}_{i+1}-{SA}_{i}}{\Delta T}=\frac{{SP}_{i+3}-3{SP}_{i+2}+3{SP}_{i+1}-{SP}_{i}}{{\left(\Delta T\right)}^{3}}$$

To accommodate noise in position estimate, we used a Savitsky-Golay smoothing filter after each discrete difference calculation. The feature for Sacrum Jerkiness is just the average magnitude of jerk for the sequence, given by summing the absolute value of *SJ*_*i*_ and dividing over the number of frames, *N*.

$$\text{Sacrum Jerkiness}=\underline{SJ}=\frac{1}{N}\sum_{i=1}^{N}\left|{SJ}_{i}\right|$$

The acceleration of the ankles or wrists can be derived using the finite differences method above, applied specifically to the joints in question. Further, using the position in the Y-dimension of the ankles, *Y*_*Rankle,i*_ and *Y*_*Lankle,i*_ the floor height can be taken from the minimal value of either ankle. The Ankle Height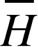 can be taken by subtracting this value from the ankle positions at each frame to get per-frame height *H*_*i*_ and averaging across all frames in the sequence.

$$\text{floor}=\min_{i}\left(Y_{R-ankle,i},i\right)$$

$$H_{i}=\frac{Y_{L-ankle,i}+Y_{R-ankle,i}}{2}-floor$$

$$\text{Ankle Height}=\underline{H}=\frac{1}{N}\sum_{i=1}^{N}H_{i}$$

Given sacrum position *SP*_*i*_, for each non-sacrum joint *j*, let **P**_*ji*_ be the 3D position vector of the joint at the *i*th frame and **V**_*ji*_ be its velocity vector. By subtracting *SP*_*i*_ from **P**_*ji*_ we get **R**_*ji*_, the position of each joint relative to the sacrum. The angular momentum **L**_*ij*_ for the *j*th joint at the *i*th frame is the cross product of **R**_*ji*_ and |**V**_*ji*_|. We get mean angular momentum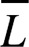 by summing and dividing **L**_*ij*_ over the number of joints *J* and frames *N*. By looking at the angular momentum in only the X and Z dimensions, we derive a dancer’s horizontal rotation.

$$R_{ij}=P_{ij}-SP_{i}$$

$$L_{ij}=R_{ij}\times \left|V_{ij}\right|$$

$$\text{Mean Angular Momentum}=\underline{L}=\frac{1}{J\times N}\sum_{j=1}^{J}\sum_{i=1}^{N}L_{ij}$$

Finally the distance **D**_*ji*_ of each joint *j* from the sacrum at frame *i* is given by the magnitude of the relative position vector **R**_*ji*_ shown above. We get mean expandedness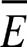 by summing and dividing **D**_*ji*_ over joints and frames.

$$D_{ij}=\left|R_{ij}\right|$$

$$\text{Mean Expandedness}=\underline{E}=\frac{1}{J\times N}\sum_{j=1}^{J}\sum_{i=1}^{N}D_{ij}$$

**FIGURE 2:**
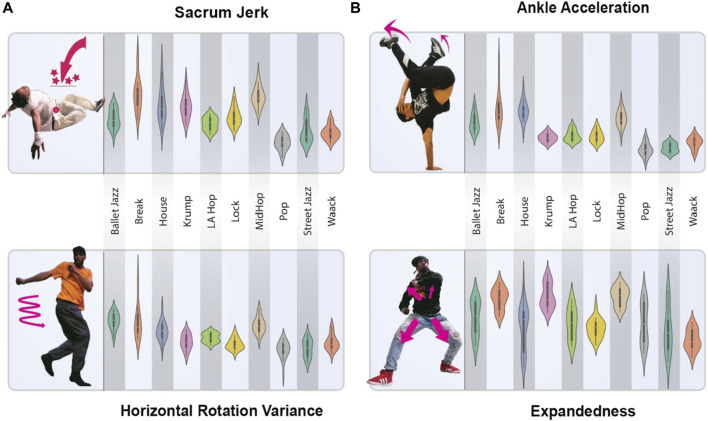
Visualization of Four Selected Dance Movement Features. The **(B)** show violin plots of four representative features derived from our dataset: Expandedness, Sacrum Jerk, Wrist Acceleration, and Bounce Regularity. The **(A)** present images of a dancer marked to illustrate the geometric interpretation of each feature. Each plot reflects distributions across the ten studied dance genres, showing the range and concentration of each feature value for each genre. The plots reveal that while the genres differ along these dimensions, there is significant overlap in the feature distributions, highlighting the complexity of genre-based differences in movement. These abstract features serve as the input for our genre classification model, capturing subtle aspects of movement to successfully distinguish between dance styles. Images of dancers provided by TheFlavorContinues.org.

**FIGURE 3:**
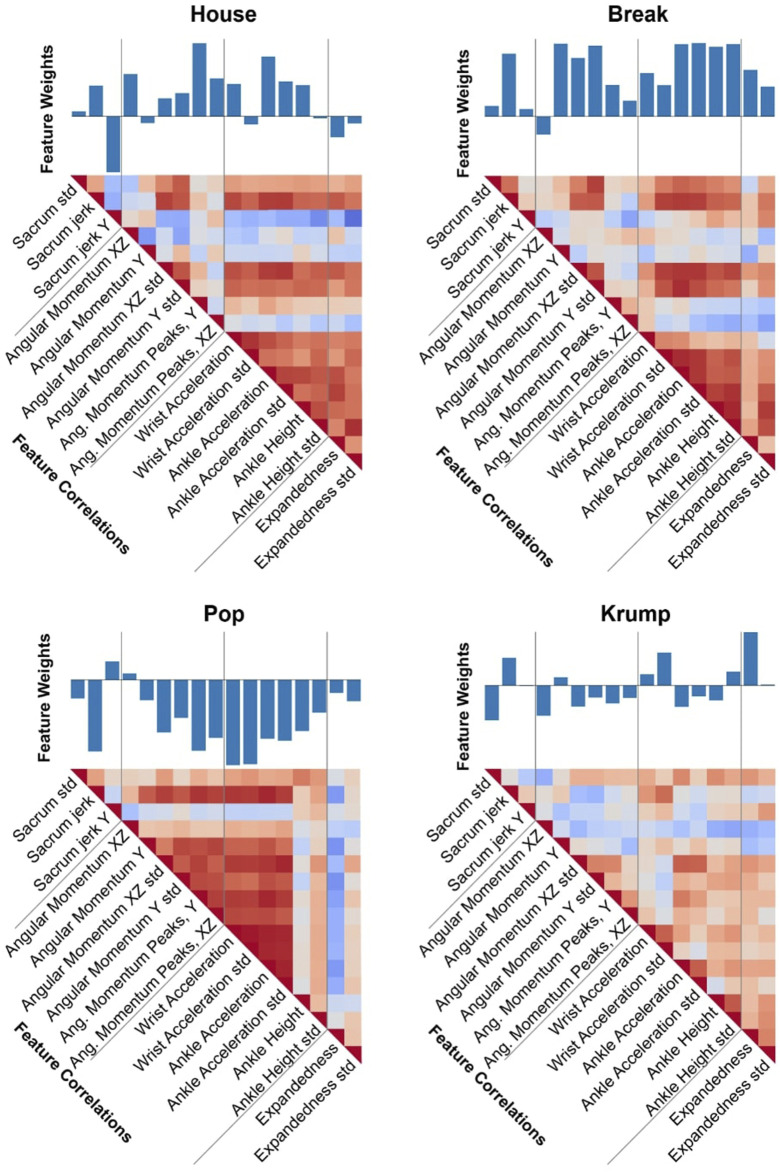
Feature-based comparison of dance genres. Each of the four subfigures corresponds to a different genre and comprises a bar plot and a correlation matrix. The bar plot depicts the 17 feature value averages for the genre. The correlation matrix visualizes how these features correlate with each other within the genre. These form a unique “fingerprint” that characterizes the style of the dance genre. It is evident that the individual features, while not perfectly distinctive on their own, collectively differentiate the genres to a significant degree. For example, Break has significantly higher values than Krump for most features relating to angular momentum, and the features relating to extremities are more highly correlated for Break than for Krump.

We visualize the spread of four of these features across all ten genres in violin plots (Figure 2), illustrating some of the characteristic differences among the genres while also showing that the genres’ exhibition of these features overlaps substantially, meaning one or even several of these measures together will be insufficient to identify the genre of a piece. We also visualize the correlation of these features with one another (Figure 3), further depicting the ways these genres differ and pointing to the way our model can use various of these features in combination to make accurate genre predictions.

### 3.3 ML classification pipeline

**FIGURE 4:**
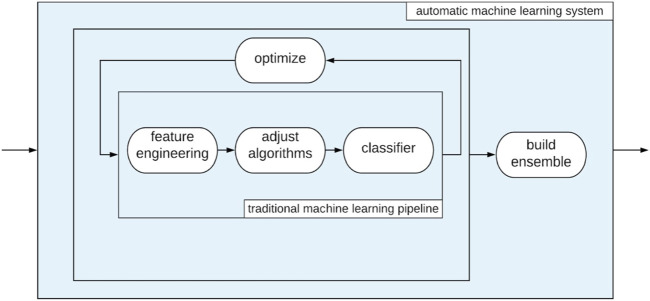
Automated machine learning through Auto-sklearn provides an end-to-end process for training performant ML models.

In order to train ML classifiers that utilize our handcrafted features, we use auto-sklearn (Feurer et al., 2015; Feurer et al., 2020), an automated machine learning framework that provides an objective end-to-end process for feature pre-processing, model selection, and model optimization (Figure 4). Auto-sklearn operates by first selecting promising initializations of model hyperparameters (i.e., parameters that control the model’s training process), then adjusting model hyperparameters using Bayesian optimization. Automatic selection of models and hyperparameters yields slightly better performance than hand tuning in most cases, and helps prevent overfitting through user bias (e.g., a preference for a particular model or knowledge of the test set data) (Saeb et al., 2016; Hosseini et al., 2020; Shen et al., 2020). By using an automated machine learning framework, we ensure that our dance genre classification models are both performant and robust. This process can be represented as follows:

f:Θ→R

$$P\left(Y|X,\Theta \right)$$

multiclass log loss *f* = $-\frac{1}{M}\sum_{m=1}^{M}\sum_{k=1}^{K}y_{mk}$ log (*p*_*mk*_)

$$\hat{y}=\sum_{i=1}^{M}w_{i}h_{i}\left(X\right)$$

The approach takes a probability distribution *P* that estimates model performance on the target variable (genre) *Y*, given input *X* (features) and hyperparameters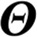 and optimizes the hyperparameters according to objective function *f*, which is the multi-class log loss function. In *f*, M is the number of instances (sequences), K is the number of classes (10 genres), *y*_*mk*_ is a binary indicator of whether *k* is the correct class label for instance *m*, and *p*_*mk*_ is the predicted probability that instance *m* belongs to class *k*. (Further details on the optimization process can be found in Feurer et al., 2020). This process results in an ensemble of models, so that a weighted sum of the predictions of these models is used for the final prediction. Ensemble prediction 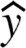
is given by the above sum where *M* is the number of models in the ensemble (20 in our case), *w*_*i*_ is the weight of the *i*th model and *h*_*i*_ is the prediction function of the *i*th model.

We split the data into separate train and test sets to build and evaluate our classifier, taking a specialized approach to handle the difference between “Advanced” and “Basic” movement sequences (see above, Section 2.1). We wanted to evaluate the classifier’s performance on “Advanced” sequences only, so we constructed a test set of 103 Advanced sequences and a training set consisting of 96 Advanced Sequences and 1199 Basic Sequences. There was a nearly even distribution of genres in the test set (mean 10.3, SD .82). Having stratified the data and set up the training and testing phases, we then applied our automated machine learning pipeline to create our ensemble of classifiers. To contextualize the performance of our ensemble model, we compared it against two well-known, simpler machine learning techniques: a perceptron and a ridge classifier. This comparison, conducted without fine-tuning these simpler models, is designed to benchmark our model’s performance and to speak to the complexity of the genre classification task.

### 3.4 Human subjects study

**FIGURE 5:**
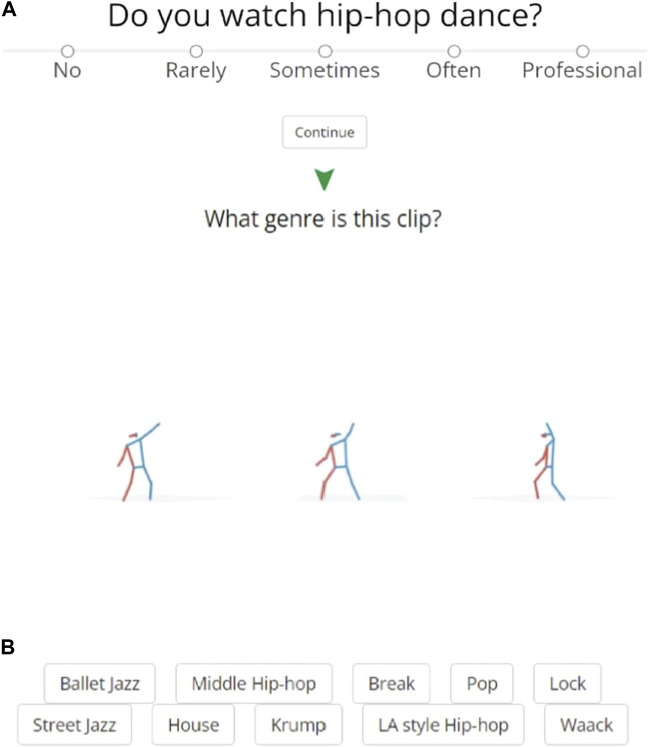
Human Subjects Study Interface. The **(A)** presents one of the initial survey questions used to gauge participants’ experience. The **(B)** presents a snapshot of the task interface that participants interacted with during the experiment. It shows a stick-figure representation of a dance sequence from our dataset, followed by the ten possible genre options for the participant to choose from.

In order to compare our model’s performance with human ability, we devised a straightforward human subjects study. We created an online experiment which we shared on social media platforms and with personal contacts, targeting individuals with experience of Hip Hop Dance. The experiment begins with a few short questions to gauge the participants’ familiarity with the domain. Participants are then tasked with watching sixteen “Advanced” sequences from the test set we used to evaluate our model. They were presented with three simultaneous views of the same stick-figure dance sequence with no music, which would repeat upon completion, and they could take as long as they wanted to identify the genre of the dance from a list of the ten possibilities (Figure 5). We sourced these video sequences from the AIST++ website. Our web application hosted *muted* versions of these videos, where audio had been removed to avoid providing clues in the musical style or beat. In order to assess human participant performance on this task, we randomly selected videos from our test set for human labeling. We recorded participants’ genre guesses for each sequence, as well as metadata such as how long it took the subject to make a decision, and the presentation order.

Our informal recruitment process and minimal survey questions serve to ensure participant confidentiality and lower costs, however they also have important limitations, especially in ascertaining the participants’ expertise levels. The short, five-option questions about participant’s familiarity with Hip Hop Dance leave substantial room for interpretation, and we did not find they correlated well with performance. Feedback we received from individual participants confirmed that the task posed a significant challenge even for our more experienced subjects, and suggested that participants without much background felt they were almost guessing at random, and were liable not to complete all sixteen guesses (in which case their responses were not recorded). This and our targeting of subjects make it likely that most of our results from this experiment come from participants with moderate to high experience viewing or participating in Hip Hop Dance. In order to provide a conservative–though not exhaustive–estimate of moderately experienced human-level performance, our analysis focuses on those participants who indicated at least a “sometimes” level of engagement with hip-hop dance (comprising 50 subjects, for a total of 800 responses).

### 3.5 Final statistical analysis

To evaluate our model’s predictive performance for classification across the ten dance genres, we primarily rely on accuracy scores. We also report precision (for sequences where the classifier predicted genre G, how many are correct?), recall (for sequences truly of genre G, how many are correctly classified?), and F1 scores (a balanced representation of both precision and recall). We also provide confusion matrices for both our ML model and the perception study, allowing for visual comparison of the overall performance of our machine and our human participants.

In addition to evaluating predictive performance, we also used dimensionality reduction and feature importance techniques to further interpret and validate our model. Specifically, we employed Latent Semantic Analysis (LSA), a technique that allows for the extraction of underlying “concepts” or “topics” from large volumes of data, typically textual data. At its core, LSA employs a form of matrix factorization (specifically, singular value decomposition (SVD)) to create a reduced-dimension representation of the original data. This allows us to represent the featurization of the genres in a 3D space, where each dimension corresponds to some meta-feature abstracted from our features, providing a visualization of similarities and differences amongst the genres. Furthermore, we rely on Shapley Additive Explanations (SHAP), to investigate feature importance (Lundberg and Lee, 2017). SHAP is a method inspired by cooperative game theory for determining which features are most influential in the model’s decisions (the “players” in the game are the features, and the outcome is the classification). This provides for better understanding of how particular genre classifications were influenced by the features. The details of these analyses and the insights they provided are presented in the Results and Discussion sections below.

### 3.6 Limitations

Our methodology is importantly limited by the fact that the assignment of movement sequences to a genre is not objective. Further, certain forms of movement are not plausibly assignable to exactly one genre, as genres borrow from one another or independently come up with similar movement forms. When we see a jumping cross step we may ask, is it a toprock from Break or a groove from Middle Hip Hop? There may be no context-free answer; nothing in the spatio-temporal aspects of the move may be able to fully determine the genre. The creators of the AIST++ dataset were careful and well-informed, but using their labels as “ground truth” amounts to a substantial idealization. In particular, experts we consulted with felt that the Street Jazz sequences were not as representative as others, and said that, while they could distinguish the LA- and Middle Hip Hop varieties after a few examples, these are not widely recognized names for genres. One implication of this is that 100% accuracy is not a realistic goal in a task like ours. Further, some of what a move expresses comes from its interaction with the music and the local context, which our methods do not capture. To an extent this also speaks to the power of our approach, since it is able to discern among genres whose definitions depend first and foremost on the contexts and aims of dance communities, rather than on details of position and motion.

Our human subject study is also limited in how it represents the ability of human observers to recognize genre from movement alone. Firstly, the issue of quantified genre affects this part of our study as well, since a single “best answer” is not always well-determined. Further, our process of recruiting via online solicitation and the minimal information we asked subjects to provide do not allow for a close assessment of how human performance on this task varies with experience, and how experienced our sample was. Without a doubt, given different backgrounds in watching and practicing dance we would expect different performance on our task, but our study was not sufficiently powered to assess this kind of difference. Rather we were only able to approximately gauge performance of people with “moderate or more” experience observing Hip Hop. Nonetheless, we think this provides a useful starting point for comparison. It is our hope that future work can look in more detail at this special human perceptual ability and its sources of variation.

## 4 Results

Our model was trained on a set of 96 Advanced Sequences and 1199 Basic Sequences from the AIST++ dataset of 10 genres of Hip Hop dance (see above, Section 3.1). We designed a custom feature set that extracted measurements of 17 abstract qualities of human movement (Section 3.2). We used auto-sklearn, an automated machine learning framework, to create our classifier (Section 3.3).

We primarily compared our model’s performance to that of human evaluators, who, with some or more exposure to Hip Hop dance, achieved a classification accuracy of 38% across 800 attempts. This baseline highlights the nuanced nature of dance genre classification, a task that even moderately experienced viewers find challenging. Additionally, we benchmarked against two straightforward machine learning algorithms: a perceptron and a Ridge Classifier, which achieved accuracies of 46% and 48%, respectively, without any fine-tuning. A Ridge classifier relying only on expandedness (the feature our model found most important, as we discuss below) achieved 20% accuracy. These figures serve as a baseline and underscore the complexity of accurately capturing and classifying the subtleties of dance movements.

Our auto-sklearn ensemble classifier significantly outperformed these baselines with an accuracy of 76%, rivaling the best human evaluators in our study (see Table 1). This performance can be compared indirectly to the findings of Castro et al. (2018) using the “Let’s Dance” dataset (see related work - a CNN-based approach relying on 2D RGB data from 10, more-readily distinguished genres, 70% accuracy). Our results demonstrate the efficacy of our approach to distinguishing these Hip Hop Dance genres, and further suggests the potential for applicability to other forms of dance and domains of movement.

**TABLE 1:** Performance metrics of the machine learning model (an ensemble chosen by automated machine learning pipeline) and of the sample of human subjects who indicated moderate or more familiarity with Hip Hop Dance.

Model results	Precision	Recall	f1-score	Support
**Ballet Jazz**	0.83	1.00	0.91	10
**Break**	0.83	0.56	0.67	9
**House**	1.00	0.73	0.84	11
**Krump**	0.73	1.00	0.85	11
**LA Hip Hop**	0.67	0.91	0.77	11
**Lock**	0.71	0.45	0.56	11
**Mid Hip Hop**	0.71	0.91	0.80	11
**Pop**	0.78	0.70	0.74	10
**Street Jazz**	0.83	0.56	0.67	9
**Waack**	0.64	0.70	0.67	10
**accuracy**			**76%**	103
**macro avg**	0.77	0.75	0.75	103
**weighted avg**	0.77	0.76	0.75	103

Human results	Precision	Recall	f1-score	Support
**Ballet Jazz**	0.62	0.70	0.66	70
**Break**	0.83	0.56	0.67	71
**House**	0.32	0.32	0.32	78
**Krump**	0.62	0.50	0.55	105
**LA Hip Hop**	0.20	0.19	0.19	86
**Lock**	0.39	0.33	0.36	85
**Mid Hip Hop**	0.30	0.30	0.30	84
**Pop**	0.27	0.31	0.29	71
**Street Jazz**	0.28	0.35	0.30	75
**Waack**	0.26	0.35	0.30	75
**accuracy**			**38%**	800
**macro avg**	0.39	0.38	0.38	800
**weighted avg**	0.39	0.38	0.38	800

The value for overall accuracy is in bold, representing the most straightforward measure of comparison.

## 5 Discussion and analysis

Our study establishes a novel method of characterizing significant aspects of Hip Hop Dance movement in a quantitative, human-understandable form. The 17 measures we used capture intuitive features of full-body movement, and the strong performance of our model in distinguishing among the ten genres indicates that the artistic expressions of different Hip Hop Dance styles depend significantly on these features. As different communities have innovated on existing styles and converged on movement genres that suit their expressive aims, they have explored and settled on different ways of combining the aspects of movement we looked at. This confirms first of all that Hip Hop Dance has evolved in a way where genres have differentiated themselves according to characteristic movement dynamics, suggesting that these genres should not primarily be understood in terms of the associated attire, music, or setting, but in the feel and look of the movements themselves.

**FIGURE 6:**
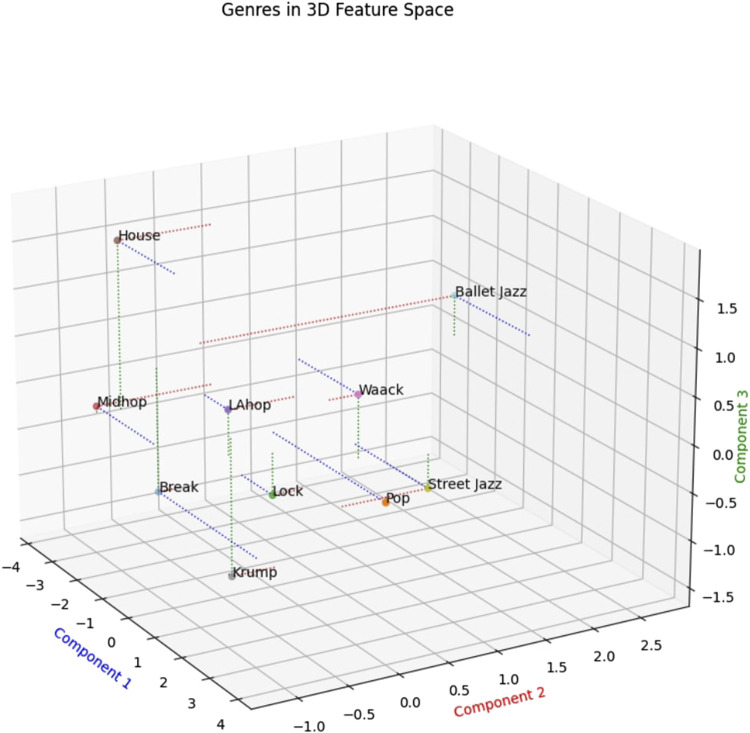
Similarity between genres derived from our features, reduced to three dimensions using LSA. As we describe in the discussion section, the first component correlates with up-and-down movement or “bounce,” the second component with momentum, and the third component with repetitiveness or regularity of motion.

Given the effectiveness and interpretability of our method, it can provide a basis for linking aspects of a dance’s kinematics to its personal and cultural connotations - grounds from which to build bridges between the movements and their meaning. To begin to uncover the high-level aspects of genre to which our features relate, a good approach is to visualize and analyze the similarity-space populated by the dances in this dataset, as defined by our features. We do this using Latent Semantic Analysis (LSA), a technique for reducing the dimensionality of our encoding from 17 down to 3, allowing us to plot the genres in a 3D space (Figure 6). LSA involves a method of matrix factorization called Singular Value Decomposition (SVD), wherein the resulting singular values allow for a compressed representation of the data, made up of features abstracted from the larger-dimensional representation of the initial encoding. By analogy to text, one could consider each genre as a document and each movement feature as a word that might show up in any document, and LSA as abstracting out the most prevalent semantic features from the relationships among words (movement features) in the different documents (genres).

The relationships between points in this similarity-space derived from our features align with several basic characteristics of these genres that people experienced with them would find intuitive (characteristics that are not the focus of research we are aware of, but are rather largely drawn from our experience and discussions with various practitioners and researchers). For instance, the fact that Krump, House, and Ballet Jazz are farthest from the center of the space and each other agrees with the sense that these three are among the most different from one another in their overall look and feel. Also that fact that LA Hip Hop sits closest to the center of the space fits with the thought that this genre is the most hybrid, historically and aesthetically. LA Hip Hop is the genre that most commonly appears on popular music videos and stages, its typical audience probably has more non-dancers than the rest of these genres, and it draws extensively on elements from several of the other genres. Because this similarity space seems to correspond well with some rudimentary facts about these genres, inspecting it further and bringing to bear more knowledge about these genres offers a way to deepen our understanding of these dance styles and how they may have evolved.

First, by considering some characteristics of the genres that mark off the outsides of the space, we can get a qualitative sense of what the 3 dimensions of the space represent, allowing for insights about how different volumes of the space relate to the history and meaning of different Hip Hop styles. Component 1 appears to track vertical movement or bounce (with higher bounce being negative); House, Break, and Middle Hip Hop typically have the most regular, global up-and-down motion, whereas Krump tends to keep a level center-of-gravity for extended periods. Component 2 appears to track overall momentum, with the slower, lighter styles of movement at the positive end. Component 3 appears to track rhythmic regularity, with the steady, cyclical movements of House (and to an extent, Ballet Jazz) at the top and the more abrupt and erratic genres at the bottom.

We can use this similarity space to support certain hypotheses about how Hip Hop Dance has evolved and what it might explore in the future. First note that the Hip Hop genres that emerged first among these are Break and Lock. Ballet Jazz originates considerably earlier, well before the “Hip Hop” moniker appeared, with roots in the much older form of Ballet, and is thus the least connected to the broad category of Hip Hop Dance (Guarino and Oliver, 2014). The distances to various genres from Hip Hop’s origin point does not seem to correlate well with chronological or geographical appearance; LA Hip Hop is the most recent and is mainly located across the US from Hip Hop’s origins, whereas Pop arrives on the scene fairly early and also on the East Coast. This lets us see Hip Hop Dance’s evolution as having modified the movement dynamics of earlier styles in a way that afforded the artistic aims of the communities that formed these genres, beginning with Break and winding its way around the space of possibilities.

One conclusion invited by this analysis is that Hip Hop Dance communities are unlikely to seek styles with more overall movement (Component 2) than the earliest genres. House and Krump become widely recognized forms later than Break, and while they differ substantially they appear comparable in terms of Component 2. Perhaps these styles already approach a level of overall movement that pushes the human body to certain physiological limits.

This similarity space raises a question about the large, unoccupied volume in the top-center of the space. A dance in this area would appear to be halfway between Krump and Ballet Jazz in its overall energy and bounce, but with substantial rhythmic regularity comparable to House. Perhaps there is an existing style outside of the dataset that exhibits this combination of features, but we are not aware of it. Or perhaps this is a part of the similarity space where the style of movement fails to be aesthetically desirable or interpretable, though we doubt it. A plausible hypothesis is thus that a new genre has yet to coalesce in this region. We hope in future investigations to identify dance sequences that populate that part of this space and try to discover what, if anything, might hold some of them together as a distinctive, emerging kind of dance.

**FIGURE 7:**
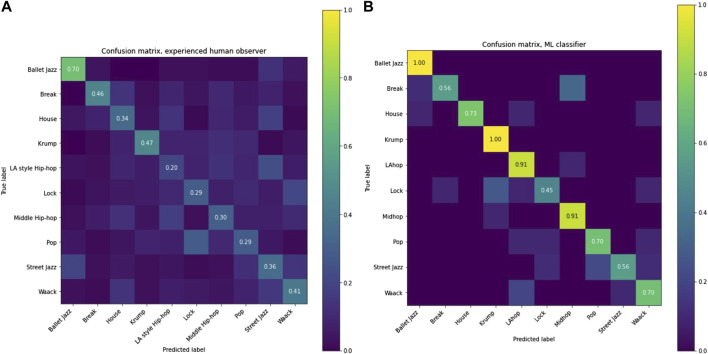
Confusion matrices for human participants **(A)**and classifier **(B)**.

By comparing the performance of our computational classifier to our sample of human genre-evaluators, we can inquire into the extent to which our model might explain how people can tell genres apart. The confusion matrix (Figure 7) shows how often a prediction erred in each genre, and which genres were erroneously guessed in those cases.

Human evaluators found Ballet Jazz easiest to classify by a considerable margin, followed by Krump, which was closely followed by Break. Ballet Jazz and Krump were also the two genres on which the model performed perfectly, and they were separated by the greatest distance in similarity space. This suggests a substantial degree of overlap in the features that served prediction by our model and the characteristics that allow people to distinguish movement styles, further supporting our claim that the features we chose capture aspects of dance movement that are salient to human dancers and appreciators of dance.

On the other hand, several differences in relative performance point to the fact that people are likely relying on information that the model is not picking up. For example, the model’s performance was relatively lower (though absolutely higher) on Break, compared with humans. This could be partly because breakdancing sequences often involve particular floor movements that can make a human observer highly confident in the genre, but that do not dramatically shape the measures of the entire sequence that our model uses. Another notable difference in performance was that our human sample found LA Hip Hop the most difficult to classify whereas the model performed relatively well on this genre. Again, this could be due to people making judgments based on a small section of a given sequence - one or two movements that seem especially indicative of a genre - where this could lead them astray with LA Hip Hop since it incorporates various elements from other genres. In future work we would like to try to better understand human genre judgments both through closer examination of human performance and by refining our machine-learning pipelines to integrate its predictions over many small time-windows comprising a dance sequence.

**FIGURE 8:**
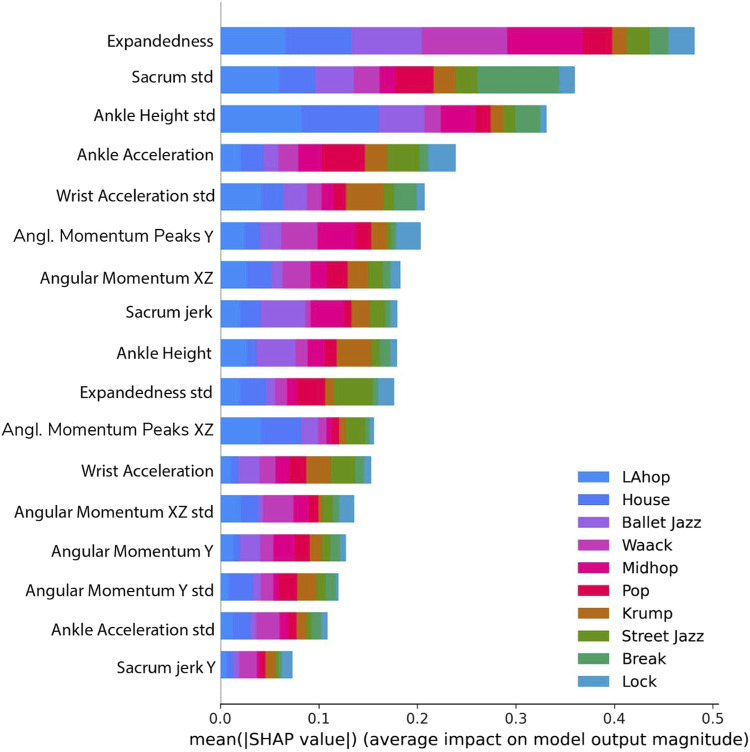
Influence of features on model output, colored according to genre.

**FIGURE 9:**
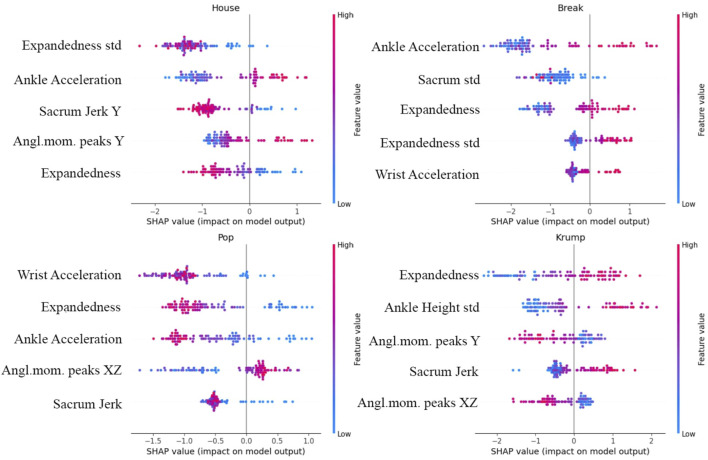
Top five most influential features for House, Break, Pop, and Krump, where the color of each dot reflects the value of the feature for one sequence, and its position reflects the degree of positive or negative impact on the model’s output. The general importance of expandedness is evidenced, as are various distinguishing aspects of these four genres, such as the relative significance of sharp movements and movements of the extremities.

By examining how our model’s performance depends on the features and relations among them, we see which features are most closely associated with differences in dance style and inform further reflection on expressive human movement. We use SHAP to investigate the relative contribution of different features to the model’s classification, in general and with respect to particular genres (Figures 8, 9). Expandedness stands out as the most important feature on average, and one of the most important features for many of the genres (with its standard deviation also playing a significant role). This was one of the first measures we thought to analyze, and the SHAP results support the idea that narrative and aesthetic content of movement registers significantly in how expanded a dancer tends to be, and how this changes over time. These results also confirm that sacrum movement is a key feature (2nd overall), which we thought was likely since the sacrum affects so much of the body’s kinetics as well as the mood of a dance. We were somewhat surprised that the standard deviation of ankle height was so high on the list of important features (3rd overall), and we are led to believe that this feature works well to differentiate varieties of footwork. It was also not clear to us initially that the model would need to rely so substantially on so many features. If even a few of these features are removed performance begins to drop considerably. This speaks to the complexity of the way kinematics vary across genres to enable the complex range of expressions embodied within each of the genres.

One kind of feature we were surprised to find did not especially contribute to our model’s ability to distinguish genre; our attempts to measure rhythmic uniformity. We assume changes in angular momentum to some extent correspond with the musicality of movement, as the dancer’s rotational motion should tend to accelerate and decelerate alongside certain repetitive aspects of the music. This led us to think that a dance sequence that is quite rhythmically uniform - matching an unchanging beat through the sequence - should have similar values for angular momentum in regular intervals, as the dancer repeatedly hits the same beat in a similar way. In that case the autocorrelation of angular momentum (a measure of the degree of similarity between a given time series and a lagged version of itself over successful time intervals) should exhibit prominent peaks at this regular interval, for rhythmically uniform sequences, and not so many peaks for more sporadic, or rhythmically variable sequence. However, we added 3 additional features to the set of 17 discussed above, a count of prominent peaks of the autocorrelation along each of the three spatial dimensions of angular momentum, and we found that these were the 3 least-important features in the set according to SHAP, and that model performance improved only marginally. Noting that the third component of our LSA-based dimension-reduction of our features appears to correspond with rhythmic regularity, one reasonable interpretation is that the combination of the other 17 features substantially constrains the rhythmic uniformity of a sequence.

In addition to shedding new light on these forms of Hip Hop Dance and the significant aspects of movement, our approach can readily be adapted to learn about other domains of movement and to develop applications that deal with full-body movement. The variations that distinguish these genres of Hip Hop Dance are subtle, as they must be to allow dancers within each to explore a broad range of artistic expressions. This is evidenced by the fact that a person requires substantial experience to tell these genres apart at all, and even given moderate experience people often fail to accurately recognize the genre from movement alone. The nuanced nature of distinguishing these movement forms is further supported by the fact that the perceptron and ridge classifier had worse than 50% accuracy, and that using only one or few of our features yielded much poorer performance (20% for a Ridge Classifier using expandedness). Therefore we think it likely that the aspects of movement that enable our model to distinguish these genres could provide insight into significant categories of movement in other styles of dance, in athletics, and in rehabilitative medicine. If indeed our computational kinematics can generalize to various domains, it could be applied to develop powerful technologies that are sensitive to the nuances of human movement. Our methods could enable new advances of computationally assisted movement training and therapies for dancers, athletes, and anyone with the goal of developing certain movement abilities. Our study opens a path to creating ways for choreographers to craft pieces that intentionally blend or contrast key features, or for trainers to better instruct people in safe and effective movement. The prospect of applying our methods to a wider array of movement data and integrating them with other advances in computational and material sciences offers a host of exciting possibilities for enhancing knowledge and practices around human movement.

## Data Availability

The raw data supporting the conclusion of this article will be made available by the authors, without undue reservation.
